# Pre-Sleep Arousal and Fear of Sleep in Trauma-Related Sleep Disturbances: A Cluster-Analytic Approach

**DOI:** 10.32872/cpe.v2i2.2699

**Published:** 2020-06-30

**Authors:** Gabriela G. Werner, Sarah K. Danböck, Stanislav Metodiev, Anna E. Kunze

**Affiliations:** a Department of Psychology, LMU Munich [study institution], Munich, Germany; b Department of Psychology, University of Salzburg, Salzburg, Austria; Philipps-University of Marburg, Marburg, Germany

**Keywords:** trauma-related sleep disturbances, pre-sleep arousal, insomnia, nightmares, fear of sleep, posttraumatic stress disorder, cluster analysis

## Abstract

**Background:**

Trauma-related sleep disturbances constitute critical symptoms of posttraumatic stress disorder (PTSD), but sleep symptoms often reside even after successful trauma-focused psychotherapy. Therefore, currently unattended factors – like fear of sleep (FoS) – might play a crucial role in the development and maintenance of residual sleep disturbances. However, it is unclear whether trauma-exposed individuals exhibit different symptomatic profiles of sleep disturbances that could inform individualized therapeutic approaches and eventually enhance treatment efficacy.

**Method:**

In a large online study, a two-step cluster analysis and a hierarchical cluster analysis using Ward’s method were performed to explore subgroups among trauma-exposed individuals (N = 471) in terms of FoS, different aspects of trauma-related sleep disturbances (e.g., insomnia symptoms, nightmares, arousal), and PTSD symptoms. These variables were compared between resulting clusters using ANOVAs and Scheffé’s post-hoc tests.

**Results:**

The hierarchical cluster analysis supported 3- and 4-cluster solutions. The 3-cluster solution consisted of one “healthy” (n = 199), one “subclinical” (n = 223), and one “clinical” (n = 49) cluster, with overall low, medium, and high symptomatology on all used variables. In the 4-cluster solution, the clinical cluster was further divided into two subgroups (n = 38, n = 11), where one cluster was specifically characterized by elevated somatic pre-sleep arousal and high levels of FoS.

**Conclusions:**

A subgroup of trauma-exposed individuals with PTSD and sleep disturbances suffers from increased pre-sleep arousal and FoS, which has been suggested as one possible explanation for residual sleep disturbances. In these patients, FoS might be a relevant treatment target.

## Abstract (highlights)

- Fear of sleep is one additional, important aspect of trauma-related sleep disturbances.
- Trauma-exposed individuals can be clustered based on their sleep- and PTSD-related symptomatology.
- One subgroup was specifically characterized by increased fear of sleep and somatic pre-sleep arousal.
- Fear of sleep might be a relevant treatment target and might provide more specialized treatments with greater response rates.
- Somatic pre-sleep arousal might reflect the physiological component of fear of sleep.

During the last decade, the body of research on sleep disturbances in trauma- and stressor-related disorders, particularly posttraumatic stress disorder (PTSD), has rapidly grown. Sleep disturbances following traumatic experiences are mostly conceptualized as symptoms of insomnia (e.g., difficulties falling or staying asleep) and recurrent nightmares (Pace-Schott & Bottary, 2018), which were previously seen as secondary symptoms of PTSD (Spoormaker & Montgomery, 2008). This might be due to the fact that these types of sleep disturbances are represented in the formal diagnosis of PTSD (American Psychiatric Association, 2013). However, recent research has consistently shown that sleep disturbances are more than a mere epiphenomenon, as they appear to constitute a crucial factor in the development and maintenance of PTSD (Cox, Tuck, & Olatunji, 2017; Germain, McKeon, & Campbell, 2017; Sinha, 2016; Spoormaker & Montgomery, 2008). Furthermore, although evidence-based treatment for PTSD (Lee et al., 2016; Schnurr, 2017) often leads to significant reductions in symptoms of insomnia as well as nightmares, in contrast to other PTSD symptoms, sleep disturbances do not usually fully remit (Belleville, Guay, & Marchand, 2011; Galovski, Monson, Bruce, & Resick, 2009; Gutner, Casement, Stavitsky Gilbert, & Resick, 2013; Lommen et al., 2016; Woodward et al., 2017). Sleep-focused treatments, like *Cognitive Behavioral Therapy for Insomnia* (CBT-I) or forms of trauma-related nightmare treatments (e.g., *Imagery Rehearsal Therapy*, IRT; or *Exposure, Relaxation, and Rescripting Therapy*, ERRT), lead to stronger reductions in sleep disturbances and nightmares respectively (Casement & Swanson, 2012; Ho, Chan, & Tang, 2016), and additionally moderately reduce PTSD symptoms (Davis et al., 2011; Davis & Wright, 2007; Pruiksma, Cranston, Rhudy, Micol, & Davis, 2018). However, most studies show that even after sleep-focused treatments, sleep disturbances remain in the clinical range, especially in more severe PTSD samples (Nappi, Drummond, Thorp, & McQuaid, 2010; Swanson, Favorite, Horin, & Arnedt, 2009; Ulmer, Edinger, & Calhoun, 2011). This leads to the assumption that other factors, which are currently unattended, seem to play a role in the development and maintenance of trauma-related sleep disturbances.

One such factor is fear of sleep (FoS), which includes dysfunctional beliefs about one’s perceived safety during sleep, fear of nightmares, and maladaptive behaviors. FoS seems to develop due to two main reasons: First, traumatic experiences together with daytime PTSD-symptoms (e.g., intrusive re-experiencing) induce a feeling of loss of control, which can trigger strong feelings of helplessness and reduced trust in other people and in the world (Ehlers, Hackmann, & Michael, 2004). Yet sleep is a state where a reduced ability to monitor the environment and giving up control is inevitable (Dahl, 1996). Therefore, it is plausible that trauma survivors with PTSD might be particularly fearful of this state because they feel extremely vulnerable during sleep. Second, due to a fear of re-experiencing the traumatic event during sleep, nightmares might additionally enhance FoS (Davis, 2009; Krakow, Tandberg, Scriggins, & Barey, 1995; Neylan et al., 1998). Also, related sleep-interfering maladaptive behaviors, such as sleeping with lights on, the use of heavy blankets, exaggerated safety checking before sleeping, or delaying bedtime in order to deal with nightmares or being vulnerable during sleep, can be considered part of FoS (Pruiksma et al., 2014). As FoS is not targeted during trauma- or sleep-focused psychotherapy (Pigeon & Gallegos, 2015), it has recently been suggested as an underlying mechanism of residual sleep disturbances (Pruiksma et al., 2014).

Several empirical findings support correlational links between FoS and increased symptoms of insomnia and nightmares, as well as overall PTSD symptomatology (Huntley, Hall Brown, Kobayashi, & Mellman, 2014; Kanady et al., 2018; Neylan et al., 1998; Pruiksma, Cranston, Jaffe, & Davis, 2011). However, other factors can also influence the maintenance of trauma-related sleep disturbances. For example, traumatic experiences generally lead to a state of heightened cognitive and somatic arousal – particularly during the pre-sleep period – that might consequently induce sleep disturbances (Sinha, 2016). Furthermore, the severity of trauma-related insomnia symptoms and nightmares *per se* might be one important factor for the persistence of sleep disturbances. Finally, both difficulty maintaining sleep and nightmares have also been associated with more interrupted, and therefore fragmented, rapid eye movement (REM) sleep, which can interfere with treatment response via impaired extinction learning (Pace-Schott, Germain, & Milad, 2015; Riemann et al., 2012).

Overall, there is a need to investigate these various aspects of trauma-related sleep disturbances in order to provide additional promising treatment targets. FoS might be a particularly relevant factor influencing the maintenance of trauma-related sleep disturbances because other factors (e.g., feeling of safety during the day, sleep disturbances, and nightmares in general) are already targeted during trauma- or sleep-focused therapy (Pigeon & Gallegos, 2015). However, the role of FoS in individuals with trauma-related sleep disturbances is currently unknown. Therefore, we have investigated FoS together with symptoms of insomnia, nightmares, pre-sleep arousal, and REM sleep fragmentation in the context of traumatic experiences in a general population sample that included both healthy individuals and individuals with clinically relevant PTSD symptoms. Through the use of a cluster-analytic approach, this study aims to explore symptomatic profiles of trauma-exposed individuals on FoS, insomnia symptoms, nightmares, pre-sleep arousal, and REM sleep fragmentation, as well as PTSD symptomatology. Classifying this heterogeneous group of individuals with traumatic experiences into better-defined subgroups could help to provide more specialized treatments with greater response rates, especially with regard to trauma-related sleep disturbances.

## Method

### Sample and Procedures

Overall, 754 individuals (62% female, mean age = 48.69 years; *SD* = 14.00; range 18–92) from the German nationwide online panel PsyWeb (*N* = 12.317 in 2017; https://www.uni-muenster.de/PsyWeb) participated in the study. PsyWeb is a panel that provides information about psychological topics of common interest and offers possibilities to take part in anonymous psychological tests and studies for registered members from the general population (i.e., panel members). Panel members were contacted via e-mail by the panel organization and were invited to take part in an online survey study investigating influencing factors on sleep and sleep problems. We specifically invited all panel members, independent of existing sleep problems or previous traumatic experiences. Study participants did not receive any monetary compensation but were offered automated feedback regarding their sleep quality and depression scores after completion of the survey. Participants were included if they were 18 years or older and proficient in the German language, but were excluded from all analysis if they did not give written informed consent.

It is worth noting that the data collected in this study was also used to validate the German version of the Fear of Sleep Inventory-Short Form (FOSI-SF; Drexl, Kunze, & Werner, 2019). Both projects were preregistered specifying their different research foci and analytic approaches (Kunze, Drexl, Metodiev, & Werner, 2017; Werner, Metodiev, Drexl, & Kunze, 2017).

### Measures

The survey included several measures assessing FoS, insomnia symptoms, nightmares, traumatic experiences, PTSD symptoms, and other aspects of trauma-related sleep disturbances, like arousal and a proxy for fragmented REM sleep, with higher scores indicating increased symptomatology. Traumatic experiences and PTSD symptoms were measured by the German version of the *Life Events Checklist* (LEC-5, including the extended criterion A assessment), followed by the *PTSD Checklist for DSM-5* (PCL; range 0-80; Krüger-Gottschalk et al., 2017) if any traumatic experience was indicated by the participant. In the present sample, internal consistency for the PCL was excellent (Cronbach’s α = .95). Insomnia severity was measured via the German version of the *Insomnia Severity Index* (ISI; range 0-28; Gerber et al., 2016). It assesses difficulties with initiating or maintaining sleep as well as early morning awakenings and related worries, and there is good internal consistency in our sample (α = .84) and a clinical cut-off at 15 for moderate insomnia. Furthermore, nightmares were assessed using the German version of the *Nightmare Distress Questionnaire* (NDQ; range 13-65; Böckermann, Gieselmann, & Pietrowsky, 2014) with excellent internal consistency in the present sample (α = .91). Additionally, FoS was measured via the German version of the *FOSI-SF* (Drexl et al., 2019). The FOSI-SF contains 13 items (range 0–52) on the fear of being particularly vulnerable during sleep, fear of experiencing nightmares, fear of darkness, and related behaviors, such as sleeping with lights on. The FOSI-SF showed good internal consistency in this sample (α = .86). Further measures linked to trauma-related sleep disturbances included the German versions of the *Pre-Sleep Arousal Scale* (PSAS; range 15-75; Gieselmann, de Jong-Meyer, & Pietrowsky, 2012; somatic arousal [8 items]: α = .80; cognitive arousal [7 items]: α = .92) as well as *Nocturnal Mentations* as a proxy for REM sleep fragmentation (NMS; range 3-27; Wassing et al., 2016); however internal consistency was questionable for this 3-item scale (α = .63). Depression and anxiety were assessed for exploratory purposes using the German versions of the depression module of the Patient Health Questionnaire (PHQ-9; range 0-27; Löwe, Spitzer, Zipfel, & Herzog, 2002) and the General Anxiety Disorder Screener (GAD-7; range 0–21; Löwe et al., 2008).

### Statistical Analyses

Only participants with at least one potentially traumatic experience (according to DSM-5) – and therefore valid values for PTSD symptom severity (PCL) – were further included in the analyses. Potentially traumatic experiences were defined on the basis of the LEC-5 and the extended criterion A assessment if one of the traumatic events was personally experienced or witnessed (Weathers et al., 2013). However, if the indicated index traumatic event for the PCL did not include any of the following criterion A characteristics, the participant was assigned to the no-trauma group and excluded from further analyses. The criterion A characteristics were: Danger of life, serious injury, sexual violence, or – in the case of the death of a close family member – accident or violence. After exclusion, the remaining sample consisted of 471 trauma-exposed individuals (see Table 1 for demographic variables of the sample).

**Table 1:** Demographic Variables of the Trauma-Exposed Subsample (n = 471)

Variable		
Age	*M*	*SD*
	49.02	13.25
Female	*n*	%
	306	64.97
Marital status	*n*	%
Single	101	21.44
In relationship	95	20.17
Married	201	42.68
Divorced or widowed	74	15.71
Education	***n***	**%**
Middle school degree	55	11.68
High school degree	98	20.81
University degree	268	56.90
Vocational education	45	9.55
Other	5	1.06
Occupation	*n*	%
Student	33	7.00
Employed	319	67.73
Unemployed	13	2.76
Retired	86	18.26
Other	20	4.25
Past psychotherapeutic treatment	*n*	%
	225	47.77

All analyses were carried out using the Statistical Package for the Social Sciences (IBM SPSS Statistics, Version 24). Cluster analysis is a data-driven approach seeking to identify specific subgroups of individuals within a larger sample on the basis of shared characteristics. Specifically, cluster analyses aim to group individuals that are similar to each other on specified variables into distinct groups. In the present study, cluster analyses were used to explore different symptomatic profiles of trauma-exposed individuals. In order to identify subgroups within our sample, we first performed a non-hierarchical two-step cluster analysis. This type of cluster analysis is advantageous, as it automatically uses standardized variables and chooses the optimal number of clusters based on Schwarz’s Bayesian Criterion (BIC) and the ratio of distance measures (Schendera, 2010); in this case the Euclidian distance measure was used. The resulting cluster quality was automatically rated based on the silhouette measure for cohesion and separation. For PTSD symptomatology we used the PCL score without items referring to sleep (“PCL-”, i.e., PCL without items 2 and 20) to decrease overlap with other measures assessing sleep-relevant variables.

As non-hierarchical cluster analyses only detect main clusters, we also conducted a hierarchical cluster analysis, using Ward’s method (Ward, 1963). This method, which has been broadly used in the social sciences (Clatworthy, Buick, Hankins, Weinman, & Horne, 2005), seeks to minimize the total within-cluster variance (leading to more homogeneous subgroups) and tends to create approximately equally sized, non-overlapping clusters (Schendera, 2010). Using this agglomerative approach, each individual initially represents its own cluster and clusters then progressively merge with others (as a function of their relative distance, i.e., the squared Euclidian distance) until one cluster including all cases is formed. The ideal number of clusters was determined by inspection of the resulting dendrogram and agglomeration coefficients, where a large increase between two consecutive cluster solutions indicates an unfavorable combination of two heterogeneous clusters and should therefore be abandoned. If the dendrogram and agglomeration coefficients supported more than one cluster solution, they were all treated as final solutions and further examined. Differences in clustering variables between the resulting clusters of the final cluster solution were then explored via subsequent analyses of variance (ANOVAs) followed by post-hoc analyses. Differences in secondary and demographic variables were investigated for exploratory purposes.

## Results

### Psychometric Variables

The psychometric characteristics of the sample with regard to variables used in the cluster analyses are given in Table 2. PTSD symptomatology is reported both overall (PCL) and without items referring to sleep disturbances (PCL-), as the latter was used for cluster analyses.

**Table 2:** Psychometric Characteristics of the Trauma-Exposed Subsample

Variable	*M*	*SD*	*Range*
PTSD symptoms (PCL)	14.60	14.71	0–71
PTSD symptoms without sleep disturbances (PCL-)	13.23	13.57	0–63
Insomnia symptoms (ISI)	8.63	5.39	0–26
Nightmare distress (NDQ)	23.10	8.99	13–54
Fear of sleep (FOSI-SF)	2.07	4.39	0–35
Pre-sleep arousal (PSAS)	27.56	9.70	15–62
Somatic pre-sleep arousal (PSAS, somatic subscale)	12.56	4.51	8–32
Cognitive pre-sleep arousal (PSAS, cognitive subscale)	15.01	6.30	7–33
REM sleep fragmentation (nocturnal mentations)	10.86	5.91	3–27

*Note.* PCL = Posttraumatic Checklist; PCL- = PCL score without items 2 and 20; ISI = Insomnia Severity Index; NDQ = Nightmare Distress Questionnaire; FOSI-SF = Fear of Sleep Inventory-Short Form; PSAS = Pre-Sleep Arousal Scale.

### Two-Step Cluster Analysis

The lowest BIC (2067.80) and the largest ratio of distance (1.39) both supported a 2- cluster solution, which was automatically chosen. Cluster quality was rated as good, based on the silhouette measure for cohesion and separation. Cluster 1 was characterized by low values of all variables (“healthy cluster”; *n* = 418), and cluster 2 was characterized by high values of all variables (“clinical cluster”; *n* = 53) (see Table 3). Subsequent *t*-tests revealed significant differences between the two clusters on all grouping variables, *p*s ≤ .001.

**Table 3:** Mean Scores of Clustering Variables in Clusters Obtained by Two-Step Cluster Analysis

Variable	Healthy cluster *n* = 418		Clinical cluster *n* = 53	
	*M*	*SD*	*M*	*SD*
PCL	11.35	11.01	40.25	15.09
PCL-	10.28	10.28	36.45	14.07
ISI	7.69	4.78	15.98	4.09
NDQ	21.18	6.92	38.28	9.10
FOSI	1.06	2.00	10.09	8.27
PSAS-S	11.70	3.53	19.32	5.65
PSAS-C	13.70	5.16	25.28	4.95
NMS	10.22	5.72	15.85	4.93

*Note.* PCL is reported for descriptive purposes, PCL- was used as clustering variable. PCL = Posttraumatic Checklist; PCL- = PCL score without items 2 and 20; ISI = Insomnia Severity Index; NDQ = Nightmare Distress Questionnaire; FOSI-SF = Fear of Sleep Inventory-Short Form; PSAS-S = Pre-Sleep Arousal Scale somatic subscale; PSAS-C = Pre-Sleep Arousal Scale cognitive subscale; NMS = Nocturnal Mentations.

### Hierarchical Cluster Analysis

The dendrogram of the hierarchical cluster analysis using Ward’s method and squared Euclidian distances showed possible solutions of two, three, and four clusters (see Figure S1, Supplementary Material). There was a smaller increase in agglomeration coefficients between the 4- and 3-cluster solutions (155.73) and a larger increase between the 3- and 2-cluster solutions (525.09), indicating a stronger increase in the heterogeneity within clusters between the latter solutions (see Table S1, Supplementary Material). Therefore, the 2-cluster solution was abandoned and the 3- and 4-cluster solutions were further described.

The 3-cluster solution revealed two bigger clusters and one smaller cluster (Cluster 1: *n* = 199; Cluster 2: *n* = 223; Cluster 3: *n* = 49). One-way ANOVAs and Scheffé’s post-hoc comparisons indicated that all clusters differed significantly from each other regarding all variables (see Table 4 for descriptive values and inferential statistics). Specifically, Cluster 1 was characterized by low levels, Cluster 2 by medium levels, and Cluster 3 by high levels of all variables. Based on these results, and taking the clinical cut-offs for insomnia (ISI ≥ 15) and PTSD symptoms (PCL ≥ 33) into consideration, these clusters were named “healthy cluster”, “subclinical cluster”, and “clinical cluster” (see Table 4). In line with the two-step cluster analysis, the clinical clusters of both analytic approaches are comparable with respect to cluster size (*n* = 53 vs. *n* = 49) and mean scores of all variables.

**Table 4:** Descriptive Values and Inferential Statistics of the 3-Cluster Solution

Variable	Healthy cluster *n* = 199		Subclinical cluster *n* = 223		Clinical cluster *n* = 49		Statistics	
	*M*	*SD*	*M*	*SD*	*M*	*SD*	*F*(2, 468)	η^2^
PCL	5.36_a_	5.15	16.98_b_	12.31	41.29_c_	14.16	256.36***	.52
PCL-	4.84_a_	4.81	15.39_b_	11.58	37.45_c_	13.27	239.18***	.51
ISI	4.78_a_	2.92	10.39_b_	4.63	16.22_c_	3.92	210.29***	.47
NDQ	16.92_a_	3.32	25.27_b_	7.30	38.35_c_	9.09	260.68***	.53
FOSI	0.20_a_	0.57	2.24_b_	3.01	8.92_c_	9.11	115.86***	.33
PSAS-S	10.06_a_	2.14	13.09_b_	3.87	20.27_c_	4.84	183.94***	.44
PSAS-C	10.61_a_	2.71	16.45_b_	5.25	26.29_c_	3.42	303.46***	.56
NMS	7.23_a_	4.00	12.90_b_	5.62	16.29_c_	5.07	102.60***	.31

*Note.* PCL is reported for descriptive purposes, PCL- was used as clustering variable. Omnibus tests and η^2^ of one-way ANOVAs are reported (independent variable: cluster, dependent variables: clustering variables). Different subscripts indicate significant differences in Scheffé's post-hoc comparisons (*p* < .001). PCL = Posttraumatic Checklist; PCL- = PCL score without items 2 and 20; ISI = Insomnia Severity Index; NDQ = Nightmare Distress Questionnaire; FOSI-SF = Fear of Sleep Inventory-Short Form; PSAS-S = Pre-Sleep Arousal Scale somatic subscale; PSAS-C = Pre-Sleep Arousal Scale cognitive subscale; NMS = Nocturnal Mentations.

***p < .001.

**Figure 1:**
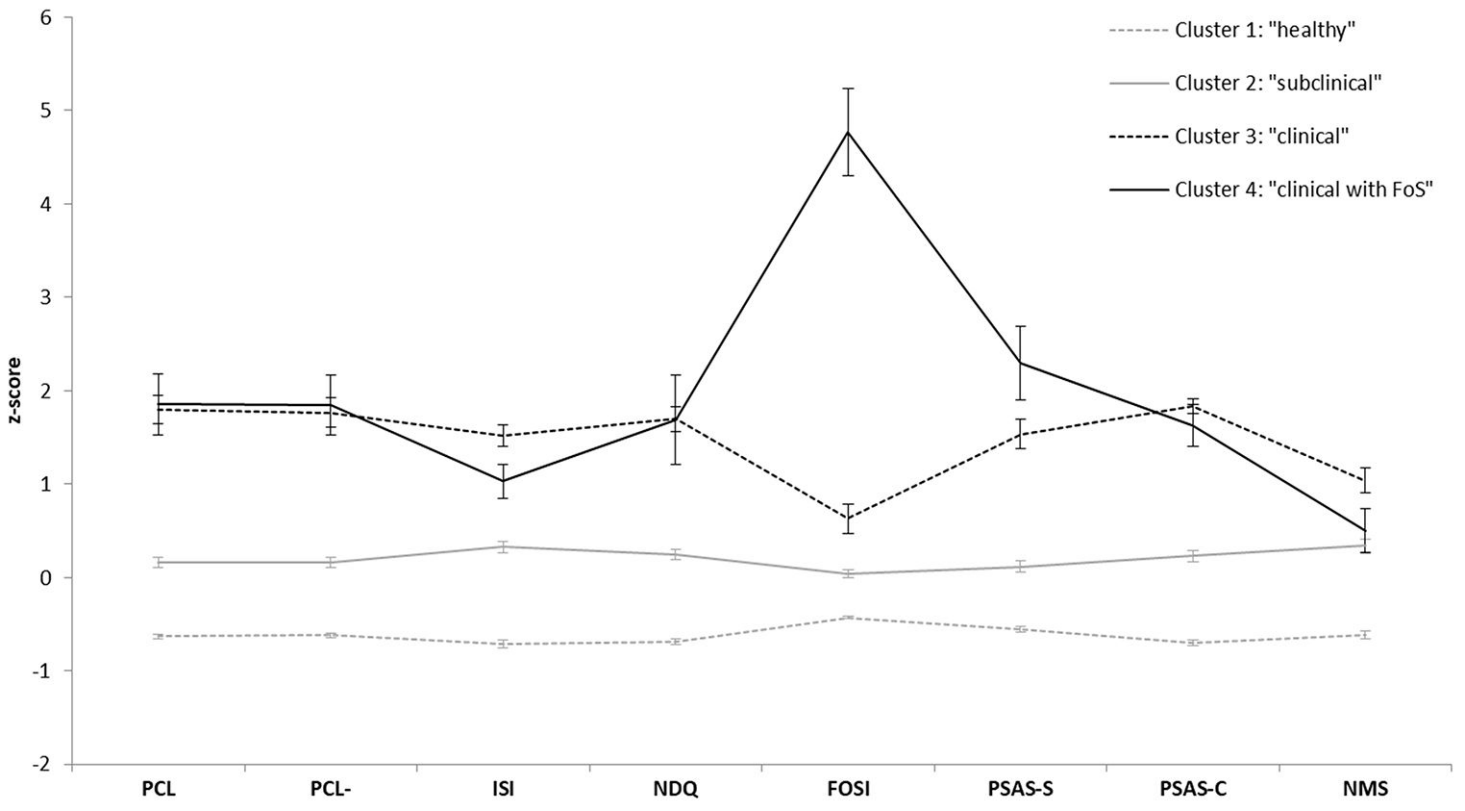
Profile of z-Scores (With Standard Error Bars) for Clustering Variables by Cluster Group *Note.* PCL scores are reported for descriptive purposes only and were not included in the analysis. PCL = Posttraumatic Checklist; PCL- = PCL score without items 2 and 20; ISI = Insomnia Severity Index; NDQ = Nightmare Distress Questionnaire; FOSI-SF = Fear of Sleep Inventory-Short Form; PSAS-S = Pre-Sleep Arousal Scale somatic subscale; PSAS-C = PSAS cognitive subscale; NMS = Nocturnal Mentations.

Regarding the 4-cluster solution, the clinical cluster was further divided into two clusters (Cluster 3: *n* = 38; Cluster 4: *n* = 11). One-way ANOVAs and Scheffé’s post-hoc comparisons were again used to explore differences between the identified clusters regarding all clustering variables as well as some additional exploratory and demographic variables (see Table 5 for descriptive values and inferential statistics). In line with the 3-cluster solution, Scheffé’s post-hoc comparisons demonstrated that both clinical clusters were characterized by significantly higher levels of all variables compared to the subclinical cluster and the healthy cluster; only nocturnal mentations did not differ significantly between one of the clinical clusters (Cluster 4) and the healthy cluster (Cluster 1), *M*_Diff_ = 0.92, *p* = .948 (see Table 5). However, comparing both clinical clusters, Cluster 4 additionally showed significantly increased levels of somatic pre-sleep arousal, *M*_Diff_ = 3.41, *p* = .033, as well as much higher levels of FoS than Cluster 3, *M*_Diff_ = 18.16, *p* ≤ .001 (see also Figure 1 for cluster group mean *z*-scores). Moreover, the amount of variance in FoS that can be explained by clustergroup membership (η^2^) was much higher in the 4-cluster solution (η^2^ = .64*)* than in the 3-Cluster solution (η^2^ = .33), while the amount of explained variance of all other variables did not differ between solutions. Therefore, Cluster 4 was named “clinical cluster with FoS”, while the third cluster remained a more general “clinical cluster”.

**Table 5:** Descriptive Values and Inferential Statistics of the 4-Cluster Solution

Variable	Healthy cluster *n* = 199		Subclinical cluster *n* = 223		Clinical cluster *n* = 38		Clinical cluster with FoS *n* = 11		Statistics	
	*M*	*SD*	*M*	*SD*	*M*	*SD*	*M*	*SD*	*F*(3, 467)	η^2^
PCL	5.36_a_	5.15	16.98_b_	12.31	41.11_c_	13.84	41.91_c_	15.90	170.58***	.52
PCL-	4.84_a_	4.81	15.39_b_	11.58	37.21_c_	13.06	38.27_c_	14.60	159.19***	.51
ISI	4.78_a_	2.92	10.39_b_	4.63	16.82_c_	3.94	14.18_c_	3.25	142.35***	.48
NDQ	16.92_a_	3.32	25.27_b_	7.30	38.37_c_	7.26	38.27_c_	14.20	173.42***	.53
FOSI	0.20_a_	0.57	2.24_b_	3.01	4.84_c_	4.37	23.00_d_	6.81	278.75***	.64
PSAS-S	10.06_a_	2.14	13.09_b_	3.87	19.5_c_	4.29	22.91_d_	5.89	127.60***	.45
PSAS-C	10.61_a_	2.71	16.45_b_	5.24	26.58_c_	2.97	25.27_c_	4.67	202.52***	.57
NMS	7.23_a_	4.00	12.90_b_	5.62	17.00_c_	5.04	13.82_a, c_	4.58	69.96***	.31
PHQ-9	12.66_a_	2.82	18.05_b_	4.83	25.11_c_	4.79	27.45_c_	5.05	150.43***	.49
GAD-7	10.08_a_	2.27	13.93_b_	3.82	20.61_c_	3.51	20.09_c_	3.78	148.64***	.49
Age	51.27_a_	13.42	48.09_a,b_	12.82	44.08_b_	12.55	44.18_a,b_	14.52	4.64*	.03

*Note.* PCL scores are reported for descriptive purposes, PCL- was used as clustering variable. Omnibus tests and η^2^ of one-way ANOVAs are reported (independent variable: cluster, dependent variables: clustering variables and secondary variables). Different subscripts indicate significant differences in Scheffé's post-hoc comparisons (p < .05). PCL = Posttraumatic Checklist; PCL- = PCL score without items 2 and 20; ISI = Insomnia Severity Index; NDQ = Nightmare Distress Questionnaire; FOSI-SF = Fear of Sleep Inventory-Short Form; PSAS-S = Pre-Sleep Arousal Scale somatic subscale; PSAS-C = Pre-Sleep Arousal Scale cognitive subscale; NMS = Nocturnal Mentations; PHQ-9 = Patient Health Questionnaire, Depression Module; GAD-7 = General Anxiety Disorder Screener.

*p < .05. **p < .01 ***p < .001.

Additionally, one-way ANOVAs and Scheffé’s post-hoc comparisons were used to explore differences between the final four clusters regarding secondary and demographic variables that were not used as clustering variables (see Table 5 for descriptive values and inferential statistics). Both clinical clusters were characterized by significantly higher levels of depression and anxiety compared to the subclinical cluster and the healthy cluster. However, comparing both clinical clusters, no differences in the levels of depression and anxiety were found. Considering age, only a difference between the healthy and the clinical cluster was found indicating higher age in the healthy cluster. Furthermore, there was a significant association between cluster group membership and gender, χ^2^(3) = 23.67, *p* < .001. Overall, clusters with higher symptom severity were associated with female gender (healthy cluster: 53.8% women; subclinical cluster: 70.7% women; clinical cluster: 84.2% women; clinical cluster with FoS: 90.9% women).

## Discussion

The present study investigated FoS together with other factors that might be important for the maintenance of trauma-related sleep disturbances in trauma-exposed individuals (i.e., symptoms of insomnia, nightmares, pre-sleep arousal, REM fragmentation, and PTSD symptoms) by using a data-driven, cluster-analytic approach. Identifying different symptomatic profiles in individuals with trauma-related sleep disturbances might help to provide more individualized treatment targets. The main analyses supported a 3-cluster as well as a 4-cluster solution: The 3-cluster solution revealed one healthy, subclinical, and clinical cluster with respective low, medium, and high scores for all variables. In the 4-cluster solution, the clinical cluster was further split into two smaller clusters. Both clusters again demonstrated significantly higher levels of all variables compared to the healthy and subclinical clusters. Additionally, one of the two clinical clusters was characterized by elevated levels of somatic pre-sleep arousal and considerably higher levels of FoS compared to the other clinical cluster. The results suggest that a subgroup of individuals suffering from PTSD is characterized by increased somatic pre-sleep arousal and FoS, which might be relevant treatment targets, particularly for these individuals.

In general, trauma-exposed individuals differ dramatically with regard to their levels of psychopathology. Empirical findings indicate that, on average, around 10% of trauma-exposed individuals demonstrate residual stress-related symptoms and subsequently develop PTSD (Hidalgo & Davidson, 2000). In line with these observations, both cluster methods in this study revealed clinical clusters whose size accounted for around 10% of the trauma-exposed sample. In the two-step cluster analysis, the clinical sample consisted of 53 (11.25%) individuals who showed PTSD and insomnia symptoms above the proposed clinical cut-offs (Bovin et al., 2016; Gerber et al., 2016). In the hierarchical cluster analysis using Ward´s method, the clinical sample consisted of 49 (10.40%) individuals, again with PTSD and insomnia symptoms above the clinical cut-off (3-cluster solution). These findings support the representativeness of our online sample with regard to PTSD symptomatology. Furthermore, 47% of the trauma-exposed sample formed a subclinical cluster with significantly higher levels on all variables (i.e., FoS, insomnia symptoms, nightmares, pre-sleep arousal, REM sleep fragmentation, and PTSD symptoms) compared to the healthy cluster. In further support of the dimensionality of the constructs measured in the present study, this cluster indicated levels of subthreshold insomnia symptoms (Gerber et al., 2016) as well as medium levels on all other variables.

In the 4-cluster solution, the clinical cluster of the 3-cluster solution was further split into two clusters. While one of these two clusters was very similar to the clinical cluster in the 3-cluster solution (i.e., clinical cluster), the fourth cluster additionally showed significantly higher levels of somatic pre-sleep arousal as well as absolute levels of FoS that were nearly 5 times higher than in the clinical cluster (i.e., clinical cluster with FoS). This cluster accounted for 22% of the clinical sample and 2% of the overall sample. However, the average scores of FoS in this cluster are slightly higher than those observed in other studies with diagnosed PTSD patients, whereas the average FoS score in the other clinical cluster is significantly lower (see Figure 1). This might indicate that, although the percentage of individuals with clinically-relevant PTSD symptoms is in line with the prevalence of PTSD in the general population, the overall symptom severity, and especially FoS, is less pronounced in this online sample, with only a subgroup demonstrating FoS values that are rather comparable to those observed in diagnosed clinical samples (Kanady et al., 2018; Pruiksma et al., 2014; Short, Allan, Stentz, Portero, & Schmidt, 2018). Although a clinical cut-off for the FOSI-SF is currently lacking, a detailed assessment of various aspects of sleep disturbances, like FoS (including whether the traumatic event took place in a sleep-related context and maladaptive sleep-interfering behaviors), could inform practitioners whether or not sleep and/or FoS should also be targeted in treatment.

Furthermore, preliminary findings on the temporal links between FoS and sleep disturbances have shown that increased FoS during a baseline period predicted worse daily sleep quality during the following week in PTSD patients (Short et al., 2018). In our sample, individuals in the clinical cluster with FoS indicated that they experience FoS once or twice per week (mean FOSI-SF = 1.77), whereas individuals in the clinical cluster indicated that they never experience FoS (mean FOSI-SF = 0.37; scale 0 = not at all, 1 = a few times per month, 2 = once or twice per week, 3 = several times per week, 4 = every night). It is worth noting that in the FoS subgroup, participants overwhelmingly indicated that they experienced the fear of loss of control and being vulnerable during sleep as often as several times per week or nearly every night. Although losing control and feeling vulnerable are cognitive dysfunctional beliefs, they are also a form of anticipatory anxiety that goes along with enhanced arousal (Davis, 2009). Accordingly, our results show that pre-sleep somatic arousal, conceptualized as various physical sensations during the pre-sleep period (e.g., palpitations, breathlessness, sweating, or muscle tension), was also significantly enhanced in the FoS subgroup (see Figure 1). Somatic pre-sleep arousal might reflect the physiological component that accompanies cognitive dysfunctional beliefs about safety during sleep. In contrast, cognitive arousal was conceptualized as more general rumination behaviors and worries about sleep disturbances as well as non-sleep-related problems and a feeling of mental activation in this study. Cognitive arousal might therefore be more characteristic of individuals suffering only from insomnia, but not in the context of PTSD, where the feeling of safety is more important than the effect of non-restorative sleep (Pigeon & Gallegos, 2015). Overall, enhanced FoS might increase sleep disturbances due to increased somatic pre-sleep arousal on the one hand, and, on the other hand, through increased engagement in sleep-interfering maladaptive behaviors. Completing this vicious cycle, there is considerable evidence supporting a perpetuating role of sleep disturbances for daytime PTSD symptomatology (Short, Allan, & Schmidt, 2017).

Trauma-focused treatments aim to differentiate between past experiences and the present situation in order to restructure dysfunctional posttraumatic cognitions with regard to safety and control (König, Resik, Karl, & Rosner, 2012). However, dysfunctional beliefs about safety during sleep are not part of standardized treatments. Consequently, anticipatory anxiety together with somatic pre-sleep arousal and subsequent maladaptive behaviors might contribute to prolonged trauma-related sleep disturbances, even after remission of other PTSD symptoms (Belleville et al., 2011). These are only theoretical considerations and research investigating the sensitivity of FoS across trauma-focused treatment is yet to be conducted. Though current sleep-focused treatments do not explicitly target FoS, promising findings have been reported recently. For example, studies using trauma-related nightmare treatments (e.g., ERRT) have reported reductions in FoS from pre- to post-treatment as well as during the follow-up assessments, together with reductions in overall sleep disturbances and PTSD symptom severity (Davis et al., 2011; Davis & Wright, 2007; Pruiksma et al., 2018). It is assumed that these treatments target mastery (“I can deal with/manage the nightmares”), which might increase a more general sense of control (Germain et al., 2004). Thus, it seems plausible that trauma-related nightmare treatments, such as ERRT, might also affect FoS. Other approaches, like CBT-I, have also shown moderate reductions in FoS after 8 weekly sessions with 29 individuals with PTSD and clinical insomnia (vs. 16 waitlist controls), although beliefs about the safety of the bed or bedroom were intentionally not targeted (Kanady et al., 2018). Given that reduced dysfunctional beliefs about sleep have been linked to better sleep in insomnia (Morin, Blais, & Savard, 2002), specifically changing dysfunctional beliefs about one’s safety during sleep and the corresponding maladaptive behaviors (i.e., FoS) might reduce trauma-related sleep disturbances. Therefore, directly targeting FoS in addition to trauma-focused and/ or sleep-focused treatment in individuals with high levels of FoS might increase treatment response, especially with regard to trauma-related sleep disturbances.

### Limitations

Some limitations must be considered when interpreting the current findings. First, although the LEC-5 and specific items regarding the index traumatic experience were used to identify trauma-exposed individuals according to PTSD criterion A as defined in the DSM-5 (Weathers et al., 2013), traumatic experiences and PTSD symptomatology were based solely on online self-report measures. Second, this is the first study to classify individuals on the basis of FoS, insomnia symptoms, nightmares, arousal, a self-report proxy for REM sleep fragmentation, and PTSD symptoms. Although meaningful cluster solutions were found, we were not able to validate cluster stability and meaningfulness of cluster membership with an external criterion. Therefore, it is essential to replicate and extend the present findings in diagnosed PTSD samples. For this purpose, we are currently collecting data in PTSD patients before trauma-focused treatment with a twofold aim: 1) to investigate whether the results of the current study also hold for clinically diagnosed PTSD samples and 2) to validate the meaningfulness of the identified subgroups by using treatment outcome as an external criterion. Third, somatic pre-sleep arousal and sleep difficulties were only measured via self-report. Especially in sleep research the use of self-reported versus objectively measured sleep is an often discussed topic. However, the subjective “sleep quality” experience seems to cover aspects that cannot be exhaustively captured via objective indices yet (see Krystal & Edinger, 2008, for a discussion on this topic). The diagnosis of insomnia disorder is currently only based on subjective complaints (e.g., Harvey & Spielman, 2011), therefore focusing on subjective indices in clinical studies is a common approach. Fourth, the 3-item measure of nocturnal mentations, which was used as a self-report proxy for REM sleep fragmentation, showed low internal consistency and was the only variable that did not consistently differentiate between the healthy, subclinical, and clinical clusters. To increase the validity of this self-report proxy, future research should include physiological measures of arousal and REM sleep fragmentation. Finally, medication and substance use as well as other sleep disturbances that might occur in PTSD (e.g., sleep apnea, parasomnias, and disruptive nocturnal behaviors) were not assessed.

### Conclusion

In sum, the data-driven, cluster-analytic approach used in this study clearly supports FoS as an important characteristic and possible additional treatment target of trauma-related sleep disturbances in individuals with PTSD. Current standard trauma-focused and/ or sleep-focused treatments seem to only moderately reduce trauma-related sleep disturbances, and residual sleep symptoms often remain. The present data support the proposition that FoS might offer an important construct involved in the development and maintenance of sleep disturbances after exposure to a traumatic event, at least in a subgroup of individuals suffering from PTSD. However, research about FoS is still in its infancy and additional studies are needed to investigate whether directly targeting FoS during treatment – particularly in PTSD subgroups with high FoS scores – might enhance treatment efficacy.

## Supplementary Materials

The supplementary materials include the dendrogram of the hierarchical cluster analysis as well as the corresponding agglomeration schedule (for access see Index of Supplementary Materials below):

10.23668/psycharchives.3089 Supplement 1 Supplementary materials to "Pre-sleep arousal and fear of sleep in trauma-related sleep disturbances: A cluster-analytic approach"

WernerG. G.
DanböckS. K.
MetodievS.
KunzeA. E.
 (2020). Supplementary materials to "Pre-sleep arousal and fear of sleep in trauma-related sleep disturbances: A cluster-analytic approach". PsychOpen. 10.23668/psycharchives.3089PMC964549336397829
