# Supplementary material for: Pre-Sleep Arousal and Fear of Sleep in Trauma-Related Sleep Disturbances: A Cluster-Analytic Approach
Source: Clin Psychol Eur. 2020 Jun 30;2(2):e2699. doi: 10.32872/cpe.v2i2.2699 (PMC9645493; doi:10.32872/cpe.v2i2.2699)
Supplement: Supplement 1 [file cpe-02-2699-s1.pdf]

Pre-Sleep Arousal and Fear of Sleep in Trauma-Related Sleep Disturbances: A Cluster-Analytic Approach

Supplementary Material

Gabriela G. Werner, PhD; Sarah K. Danböck, M.Sc.; Stanislav Metodiev, M.Sc.; & Anna E. Kunze, PhD

LMU Munich (study institution), Leopoldstraße 13, 80802 Munich, Germany.

Correspondence concerning this article should be addressed to Gabriela G. Werner, Department of Psychology, LMU Munich, Leopoldstraße 13, 80802 Munich, Germany. Phone: +49 89 2180 5297. Fax: +49 89 2180 5224. E-mail: gabriela.werner@psy.lmu.de.

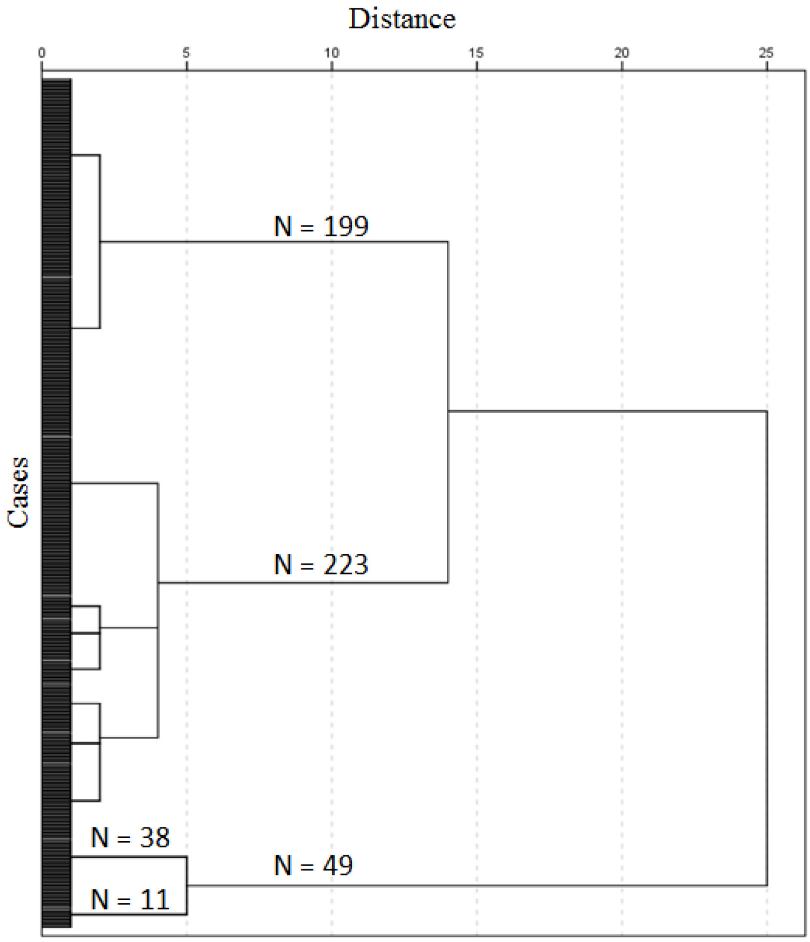

Figure S1. Dendrogram using Ward's method.

Table S1

Agglomeration Schedule Using Ward's Method

Amount of Clusters	Agglomeration Coefficients
10	1151.97
9	1198.37
8	1246.65
7	1303.17
6	1377.65
5	1514.94
4	1655.43
3	1811.16
2	2336.25
1	3290.00

Notes. Only the last section of the agglomeration schedule is provided.
